# Prácticas de autocuidado que realizan pacientes con diabetes mellitus tipo 2 de Cartagena-Colombia, 2021

**DOI:** 10.15649/cuidarte.2534

**Published:** 2023-03-29

**Authors:** Kendy Paola Madero-Zambrano, Claudia Alejandra Orgulloso-Bautista

**Affiliations:** 1 . Corporación Universitaria Rafael Núñez, Cartagena, Colombia. E-mail: kendy.madero@curnvirtual.edu.co Corporación Universitaria Rafael Núñez Corporación Universitaria Rafael Núñez Cartagena Colombia kendy.madero@curnvirtual.edu.co; 2 Universidad Santo Tomás, Bucaramanga, Colombia. E-mail: claudia.orgulloso@ustabuca.edu.co Universidad Santo Tomás Universidad Santo Tomás Bucaramanga Colombia claudia.orgulloso@ustabuca.edu.co

**Keywords:** Diabetes Mellitus, Autocuidado, Hábitos., Diabetes Mellitus, Self-Care, Habits., Diabetes Mellitus, Autocuidado, Hábitos.

## Abstract

**Introducción:**

La diabetes es una enfermedad crónica que puede causar invalidez física por sus variadas complicaciones multiorgánicas. Las prácticas de autocuidado son esenciales para el mantenimiento de la salud y la prevención de estas.

**Objetivo:**

Analizar las prácticas de autocuidado los pacientes con diabetes mellitus tipo 2 en Cartagena, 2021.

**Materiales y Métodos:**

El estudio observacional, analítico de corte transversal con 100 pacientes con diabetes mellitus tipo 2. Cuestionario (aspectos sociodemográficos, antecedentes personales patológicos y prácticas de autocuidado) que evaluó análisis univariado, bivariado y multivariado con regresión logística.

**Resultados:**

La mayoría tiene un diagnóstico de diabetes de 1-5 años (33%) y glicemia de >140 mg/dl (40%). Hubo buenas prácticas en higiene (66%), dieta (60%), sueño (50%), pero bajo nivel de actividad física (61%). Con asociación significativa de riesgo en actividad física, no tener estudio o primaria (OR: 15.44; IC 95% 2.78 85.48) y como protector en la dieta, cuando se tiene entre 41 a 56 años (OR: 0.07; IC 95% 0.00-0.60), de 52 a 72 años (OR: 0.04; IC 95% 0.00-0.42) y mayor de 73 años (OR: 0.01; IC 95% 0.00-0.28).

**Discusión:**

La población tenían un nivel bueno de prácticas de autocuidado. Es concordante con lo observado por otros autores; que determinaron que gran parte de la muestra tenían un nivel de conocimiento general alto sobre el autocuidado.

**Conclusiones:**

Las prácticas de autocuidado que realizan los pacientes con diabetes mellitus tipo 2 por lo general son buenas en cuanto a higiene, dieta y sueño, y malas respecto a actividad física.

## Abstract (en)

**Introduction:**

Diabetes is a chronic disease that can cause physical disability due to various multi organ complications. Self-care practices are essential for health maintenance and prevention of these complications.

**Objective:**

To analyze the self-care practices performed by patients with type 2 diabetes mellitus in Cartagena in 2021.

**Materials and Methods:**

An observational, analytical, cross sectional study was conducted on 100 patients with type 2 diabetes mellitus. A questionnaire (socio demographics, patients' medical background, and self-care practices) was used for univariate, bivariate, and multivariate analysis, and logistic regression analysis.

**Results:**

Most patients had a 1-to-5-year diagnosis of diabetes (33%) and blood glucose >140 mg/dl (40%). There were hygiene (66%), diet (60%), and sleep (50%) good practices, but low physical activity (61%). Self-care practices were significantly associated with physical activity risk and not having school or primary education (OR: 15.44; 95% CI 2.78 85.48). They are a dietary protector, when aged between 41 and 56 years (OR: 0.07; 95% CI 0.00-0.60), 52 and 72 years (OR: 0.04; 95% CI 0.00-0.42) and older than 73 years (OR: 0.01; 95% CI 0.00-0.28).

**Discussion:**

The population had a good level of self-care practices. It is consistent with what was observed by other authors; which determined that a large part of the sample had a high level of general knowledge about self-care.

**Conclusions:**

The self-care practices carried out by patients with type 2 diabetes mellitus are generally good in terms of hygiene, diet and sleep, and poor in terms of physical activity.

## Resumo

**Introdugáo:**

O diabetes é uma doenga crónica que pode causar incapacidade física devido a suas várias complicagóes multiorganicas. As práticas de autocuidado sao essenciais para a manutengao e prevengao da saúde.

**Objetivo:**

Analisar as práticas de autocuidado de pacientes com diabetes mellitus tipo 2 em Cartagena, 2021.

**Materiais e Métodos:**

Estudo observacional, analítico, transversal de 100 pacientes com diabetes mellitus tipo 2. Questionário (aspectos sociodemográficos, história patológica pessoal e práticas de autocuidado) que avaliou análises univariadas, bivariadas e multivariadas com regressao logística.

**Resultados:**

A maioria teve um diagnóstico de diabetes 1-5 anos (33%) e glicemia >140 mg/dl (40%). Havia boas práticas de higiene (66%), dieta (60%), sono (50%), mas baixo nível de atividade física (61%). Houve uma associagao significativa de risco em atividade física, sem educagao ou educagao primária (OR: 15,44; 95% CI 2,78-85,48) e dieta como protetora quando com 41-56 anos (OR: 0,07; 95% CI 0,00-0,60), 52-72 anos (OR: 0,04; 95% CI 0,00-0,42) e com mais de 73 anos (OR: 0,01; 95% CI 0,00-0,28).

**Discussao:**

A populagao apresentou um bom nivel de práticas de autocuidado. É consistente com o observado por outros autores; o que determinou que grande parte da amostra apresentava alto nível de conhecimento geral sobre o autocuidado.

**Conclusoes:**

As práticas de autocuidado realizadas por pacientes com diabetes mellitus tipo 2 sao geralmente boas em termos de higiene, alimentagao e sono e ruins em termos de atividade física.

## Introducción

La Diabetes Mellitus (DM) es una enfermedad crónica no transmisible, conceptualmente se define como un síndrome heterogéneo originado por la interacción genético-ambiental y caracterizada por hiperglucemia crónica, como consecuencia de una deficiencia en la secreción o acción de la insulina, que desencadena unas complicaciones agudas (cetoacidosis y coma hiperosmolar), crónicas microvasculares (retinopatías y neuropatías) y macrovasculares (cardiopatía coronaria, enfermedades cerebrovasculares y vasculares periféricas^1^.

A nivel mundial el número de personas con DM va en aumento y la Organización Mundial de la Salud (OMS) asegura que en el mundo hay cerca de 430 millones de personas que sufren esta enfermedad^2^. Además, la diabetes pertenece al grupo de las enfermedades que causan invalidez física por sus variadas complicaciones multiorgánicas, con su incremento indudable en la morbilidad y mortalidad en los últimos años, con referencia a la mortalidad la OMS establece que 3,7 millones de muertes son provocadas por la diabetes, constituyendo estos datos en un verdadero problema de salud pública^3^.

El manejo de los pacientes con diabetes depende en gran medida del nivel de comprensión de la enfermedad y la destreza ante su cuidado diario. El profesional de enfermería tiene dentro de sus funciones la atención primaria en salud con acciones encaminadas a la promoción de la salud, la prevención de la enfermedad, la recuperación y rehabilitación de los pacientes, de tal manera que sean oportunas y accesibles en todos los servicios a nivel individual, familiar y comunitario^4^.

Por lo tanto, la educación y prevención además de ser una de las actividades del equipo multidisciplinario, es en mayor medida una competencia de enfermería, puesto que está en sus manos mantener un seguimiento y apoyar a través de medidas no farmacológicas el tratamiento que lleve el paciente, ya que a través de la adquisición de conciencia sobre el cuidado propio de la salud y el trabajo arduo por estilos de vida saludable, se pueden lograr grandes cambios que garanticen la detección a tiempo y el no progreso de este tipo de enfermedades crónicas, como la diabetes que pueden puede conllevar al padecimiento de enfermedades cardiovasculares^5^.

Por consiguiente, el conocimiento y las prácticas de autocuidado son elementos esenciales para el mantenimiento de la salud y la prevención de complicaciones. De igual forma, brindar educación a los pacientes con diabetes mellitus, constituye una herramienta esencial para el enfermero frente al control y prevención de las complicaciones^6^. Sin embargo, un estudio realizado en Cuba por Soler y cols^7^, evidencio la ausencia de autocuidado en un 90%. Las dificultades se relacionan con la práctica de ejercicio físico, la nutrición adecuada y el autocontrol de la glucemia. De forma similar Arteaga y cols^8^, en su estudio realizado en México, identificaron que el nivel de aplicación de las prácticas de autocuidado fue bueno en tres dimensiones: Hábitos higiénicos (52.5%), Hábitos dietéticos (72.5%), y Sueño y descanso (67.3%). Pero en las dimensiones de Actividad física (62.5%) y Conocimiento (73.8%) fue bajo. Datos que difieren a los arrojados por Jackson y cols^9^, donde el 79,5% tenían un nivel de conocimiento general alto sobre el autocuidado. Así mismo, el conocimiento del autocuidado se asoció con el nivel de educación, el ingreso mensual y la duración de la diabetes.

En Colombia según la Organización Mundial de la Salud en comparación de los perfiles de los países para la diabetes en el año 2016 hubo un número de muerte atribuible por diabetes el cual la cifra de muestreo fue para las mujeres 30-39 años 1,450 y 70 años 2,030 y Hombres 30-69 años 1,220 y 70 años 1,350. En los cuales la prevalencia de la diabetes y los factores de riesgos conexos fueron; Diabetes con las siguientes cifras con un total de 8.0%, sobrepeso con un total de 55.8%, obesidad con cifras 20.7% e inactividad física con un total de 63.5% para ambos sexos^10^.

Una investigación realizada en Neiva-Huila, estimo que el 64,8% de las personas tenían prácticas de autocuidado medianamente adecuadas^11^. Igualmente, en un estudio realizado en Bogotá en ancianos con diabetes y factores asociados, sobre auto reporte de diabetes en la población de adultos mayores, se demostró que la evaluación de hábitos y ejercicio era del 2,9%, aún consumían cigarrillos y un 38,8% habían fumado anteriormente. En cuanto a la actividad física el 39,5% se encontraba en el tercio bajo y el 35,8% en un tercio medio y 24.6% en el tercio más alto, siendo estadísticamente significativos al compararse con la cantidad de actividad física realizada por las personas sin diabetes^12^.

A través de una búsqueda sobre la situación de diabetes en Cartagena se hallaron unas cifras establecidas por el departamento administrativo distrital de salud, el cual determina que la diabetes es una de las principales causas de mortalidad con un número de casos de 114 y una tasa de 11.12 por cada 100.000 habitantes^13^. De igual forma, la E.S.E Hospital local de Cartagena revela que para el año 2017, la incidencia de DM fue de 10.8 por cada 1000 habitantes, con un número de casos de 5.379 entre hombres y mujeres. No obstante, para el 2018, estos datos disminuyeron; tasa de incidencia de 2,6 por cada 1000 habitantes, con un número de casos de 1.326^14^.

Por lo tanto, en las personas con enfermedades crónicas como la DM son muy importante las prácticas de autocuidado frente a su estado de salud, en este proceso el profesional de enfermería juega un papel fundamental a la hora de educar al paciente sobre los comportamientos que estos deben tener en relación con su alimentación, actividad física, control de la glicemia, hábitos higiénicos de sueño y descanso; con el objetivo de modificar conductas, mejorar sus condiciones de vida y prevenir complicaciones. Desde la promoción educativa que realiza el profesional de enfermería es posible cambiar conductas, dado que estas intervenciones no solo van a estar enfocadas a la transmisión de conocimiento sobre su condición de salud, sino que debe completar también aspectos psicológicos y sociales referentes a su padecimiento^15^.

Por consiguiente, este estudio estará orientado con el modelo de promoción de Nola Pender porque trata de explicar la interacción de las personas con el entorno cuando buscan un estado de salud deseado, además destaca el vínculo entre las características personales y las experiencias, conocimientos, creencias, y los aspectos situacionales relacionados con los comportamientos y conductas de la salud que se quiere lograr, siendo así una respuesta a la forma como las personas adoptan decisiones acerca del cuidado de su propia salud^16^. Igualmente estará enfocada en la teoría de Dorothea Orem, ya que esta considera que el autocuidado es una conducta que está presente en determinadas situaciones de la vida, llevada a cabo por las personas hacia sí mismo y/o hacia su entorno, lo que le permite regular o modificar los factores que afectan su desarrollo y funcionamiento en beneficio de su vida, salud y bienestar^17^.

Los argumentos hasta aquí expuestos permitieron evidenciar la importancia de la situación anteriormente mencionada, dando lugar a enfocar el estudio en analizar las prácticas de autocuidado en paciente con DM2, debido a que este es fundamental en este tipo de patologías, ya que permite prevenir complicaciones y mantener la salud frente a la enfermedad. Además, como se evidencia previamente, esta es una enfermedad que constituye un verdadero impacto de salud pública a causa de la morbilidad y mortalidad establecida tanto a nivel internacional como nacional. Además, los resultados de esta investigación permitirán un aumento de la documentación bibliográfica que se tiene de la enfermedad, además facilitara tener una visión de las prácticas de autocuidado de los pacientes acerca de su enfermedad, para que así los entes gubernamentales involucrados en el proceso puedan implementar estrategias acordes a las necesidades reales de los pacientes con diabetes en Cartagena.

## Materiales y Métodos

Este estudio se clasificó como de tipo observacional, analítico y de corte transversal. La población estuvo constituida por personas con Diabetes Mellitus tipo 2 de viven en la ciudad de Cartagena, Colombia que cumplieran con los criterios de inclusión. No se realizó cálculo de muestra y se adoptó un muestreo no probabilístico por conveniencia^18^. Por lo tanto, participaron personas de diferentes barrios de la ciudad donde a través de un representante o líderes comunales de ellos, se comunicó la realización de la investigación para ver que habitante quería participar y cumpliera las condiciones, esto se realizó en un mes. Estos se abordaron considerando los siguientes criterios de inclusión: personas procedentes de Cartagena; personas mayores de 18 años; personas con diagnóstico confirmado de DM2. Entre tanto, el criterio de exclusión corresponde a aquellas personas con dificultad para la comunicación o con alteraciones cognitivas.

Para la obtención de la información los investigadores de este estudio aplicaron un cuestionario que fue diseñado por el Instituto Mexicano del Seguro Social, dirección de prestaciones médicas, coordinación de investigación en salud, el cual fue permitida su utilización mediante la autorización de la Dra. Elizabeth Arteaga Rojas^8^. Dicho instrumento fue validado por tres expertos en la institución que se seleccionaron por el grado académico de maestría en ciencias y por su experiencia en el estudio de las variables en cuestión. Se realizó la prueba de coeficiente de alfa de Cronbach el cual demostró una confiabilidad de 0.85 en la consistencia interna. Además, su estructura consta de cuatro componentes que permitieron cumplir los objetivos específicos, la dimensión de hábitos de higiene contiene ocho ítems, cuatro de ellos de tipo dicotómico, que se responde como ‘'si'' y ‘'no'' el cual evaluara, la frecuencia del baño corporal, lubricación de la piel, cuidado con los pies y presencia de sequedad y prurito. En el caso de la dimensión de hábitos dietéticos, este tiene catorce ítems, en el cual tres son medidos en escala Likert de tres opciones y cuatro de tipo dicotómico para explorar qué información nutricional sobre DM2 ha recibido y qué profesional de la salud se la ha proporcionado, periodicidad de la consulta al servicio de nutrición, número de comidas al día, tipo, frecuencia y cantidad de alimentos y tipo, frecuencia y cantidad de bebidas al día, así como el consumo de sal adicional en las comidas.

En el caso de la dimensión de actividad física, esta comprende cuatro ítems, dos son dicotómicos, en el cual se indaga sobre qué actividad física realiza, cuál es su frecuencia de actividad física, así como la presencia de fatiga durante la misma y finalmente, en la dimensión de hábitos de sueño-descanso, incluyen cinco ítems, con respuestas de tipo dicotómico, en los que se identificará el número de horas de sueño por la noche, dificultad para conciliar el sueño, sensación de descanso al despertar y si acostumbra a tomar siesta durante el día. La evaluación del autocuidado en general se hizo sobre el puntaje obtenido de las respuestas, el cual fue de 1 a 31 puntos en total. Los puntos de corte para determinar el nivel de aplicación de las prácticas de autocuidado fueron con base en el número total de respuestas correctas: Muy bueno (22 a 31), Bueno (11 a 21) y Bajo (1 a 10). Igualmente, de esta manera se realizó la distribución en cada una de las dimensiones de acuerdo con el número de respuestas correctas.

Los datos fueron depurados y organizados en el programa Excel versión para Windows 2010, donde investigadores realizaron validación de ésta y los datos de la investigación están disponibles en el Data- set^19^. Para el análisis se realizó en el paquete estadístico Stata/MP versión 13.0. Se ejecutó un análisis univariado donde se calcularon frecuencias absolutas y porcentajes para todas las variables. Además, con el análisis bivariado se evaluaron las diferencias entre variables cualitativas, por medio de la prueba de Chi2 de Pearson o el Test Exacto de Fisher. Para todas las pruebas se consideró una significancia estadística de valores p<0.05. Posteriormente, se realizó un análisis multivariado teniendo en cuenta como desenlace las malas prácticas de autocuidado con la regresión logística^20^ a través del método de selección intencionada de covariables donde se incluían aquellos que presentaran cambio significativo de los coeficientes >10%.

Primero se construyó un primer modelo que incluía todas las variables que en el análisis bivariado mostraron asociación con el autocuidado en pacientes con diabetes mellitus a un nivel de significancia de 0.25 y de acuerdo con lo encontrado en el marco teórico. Por su reconocida relación con el autocuidado algunas variables como sexo, estado civil y estrato socioeconómico se incluyeron en el modelo independientemente de su valor P y finalmente, se realizó el diagnóstico del modelo donde se evaluó los supuestos de normalidad y homocedasticidad de los residuos, la bondad de ajuste por medio de la prueba de Hosmer y Lemeshow y la especificidad del modelo a través de la prueba de especificación.

El estudio éticamente se fundamentó en la Resolución 8430 de 1993^21^, fue catalogado como sin riesgo debido a que no se realizará ninguna modificación de las variables biológicas, psicológicas, sociales de las personas que participen en el estudio. Las personas que autorizaron su participar en la investigación, se les proporciono un cuestionario que fue guiado por investigadores capacitados. La información que se obtuvo fue manejada con confidencialidad y no se usará para ninguna otra finalidad fuera de los objetivos de esta investigación. Igualmente, es cobijado bajo la ley 1581 de 2012^22^ que alude a la protección de los datos personales en la cual no surge ningún vínculo con el que se permita la transferencia de información a terceros. Así mismo, este estudio se sometió ante el comité de ética de la institución educativa en su acta 002 IIP 2019.

## Resultados

En esta primera parte del documento se presentan los resultados obtenidos en relación con las características más relevantes de la población:

### Caracterización de la población a estudio

El marco muestral del estudio fue de 100 pacientes con DM2 que autorizaron su participación en la aplicación del instrumento prácticas de autocuidado en Cartagena. En esta primera parte del documento se presentan los resultados obtenidos en relación con las características más relevantes de la población, donde se observa en los pacientes que en su mayoría fueron del sexo femenino en un 65,00% (65), con edades entre los 57 a 72 años 43,00% (43), el 42,00% (42) se encuentra en unión libre, un nivel educativo alcanzado de primaria en 35,00% (35), con un estrato 1 en un 77,00% (77) y quienes en un 48,00% (48) eran amas de casa.

Igualmente, con respecto a los antecedentes personales que incluyen hábitos del paciente. Se evidencio que un 94,00% (94) de la población estudiada no fuma y con el consumo de alcohol los pacientes manifestaron en un 74,00% (74) que no consume alcohol. En cuanto a los años con diagnóstico de Diabetes, se identificó que un 33,00% (33) tiene de 1 a 5 años con la patología, de igual manera aquellos que tiene >10 años de diagnóstico confirmado y manifiestan que manejan un nivel de glicemia >140 mg/dl en un 40,00% (40) (Tabla 1).

**Tabla 1:** Características sociodemográficas, personales y patológicos de la población a estudio.

Características	%	n
Sexo		
Femenino	65,00	65
Masculino	35,00	35
Edad		
Min/40 años	12,00	12
41 a 56 años	29,00	29
57 a 72 años	43,00	43
73/Max años	16,00	16
Estado civil		
Soltero	15,00	15
Unión libre	42,00	42
Casado	27,00	27
Viudo	10,00	10
Divorciado	6,00	6
Nivel educativo		
Ninguno	15,00	15
Primaria	35,00	35
Secundaria	19,00	19
Técnico/ Tecnólogo	21,00	21
Universitario	10,00	10
Ocupación		
No Trabaja	7,00	7
Ama de casa	48,00	48
Oficios varios	15,00	15
Otro	30,00	30
Nivel socioeconómico		
Estrato 1	77,00	77
Estrato 2	15,00	15
Estrato 3	5,00	5
Estrato 4	3,00	3
Consumo de cigarrillo		
Si	6,00	6
No	94,00	94
Consumo de alcohol		
Si	26,00	26
No	74,00	74
Años diagnostico DM2		
<1 año	14,00	14
1-5 años	33,00	33
6-10 años	20,00	20
>10 años	33,00	33
Nivel de glicemia		
No sabe	12,00	12
80-100 mg/dl	13,00	13
110-120 mg/dl	35,00	35
>140 mg/dl	40,00	40

### Identificación de las prácticas de autocuidado

En este capítulo de los resultados se identificaron las prácticas de autocuidado de los participantes, teniendo en cuanta los hábitos de higiene, hábitos dietéticos, actividad física, hábitos de sueño y descanso y otras variables tales como redes y grupos de apoyo y autocuidado en general. En cuanto a la primera dimensión de hábitos de higiene, los sujetos de estudio expresaron que su frecuencia de baño es diario en un 98% (98), lubrican su piel con crema para piel seca en un 65% (65), con ausencia de prurito en un 75% (75), no presentan piel seca el 61% (61), el 90% (90) si realiza cuidado de sus pies y el 82% (82) revisa sus pies en búsqueda de lesiones. Por otro lado, con respecto a los hábitos dietéticos, las personas encuestadas comen de 3-4 comidas en el día en un 83% (83), consumen alimentos entre comidas en un 65% (65), quienes agregan sal a sus comidas siempre en un 40% (40) y el 98% (98) consume alimentos naturales.

Además, el 57% (57) señalo que no llevan una dieta específica para su enfermedad, que son orientados de igual manera acerca de esta por el médico general y especialista en un 46% (46), el 51% (51) de la población de estudio no asiste a consultas de nutrición y la periodicidad a la que asisten a esta consulta, refieren que nunca lo hacen en un 42% (42). Se apreció que el 75% (75) de los participantes no consumen bebidas azucaradas y la frecuencia en que comen los siguientes alimentos: Carnes roja en un 51% (51) una vez por semana, carnes blancas un 44% (44) cada tres días, lácteos en un 38% (38) diario, pescados un 36% (36) cada tres días, verduras un 77% (77) diario, las frutas en un 71% (71) diario y leguminosas en un 46% (46) una vez por semana.

Con respecto a los resultados obtenidos en las actividades físicas, se conoció que los participantes no realizan actividades físicas en un 56% (56), mostraron que el 45% (45) realiza actividad física aeróbicas, con una frecuencia de a diario en un 28% (28), ejecutan la actividad en unos 30 a 40 minutos un 35% (35) y el 59% (59) refiere que no se fatiga cuando realiza algún tipo de actividad física. Finalmente, en los hábitos de sueño y descanso, se evidencio que un 81% (81) de la población duerme de 6-8 horas al día, un 70% (70) manifiesta que siente sensación de descanso al despertar, el 62% (62) refiere que toma siesta durante el día y, por último, se indago la presencia de insomnio en esta población en el que el 82% (82) negó padecer de este.

**Figura 1:**
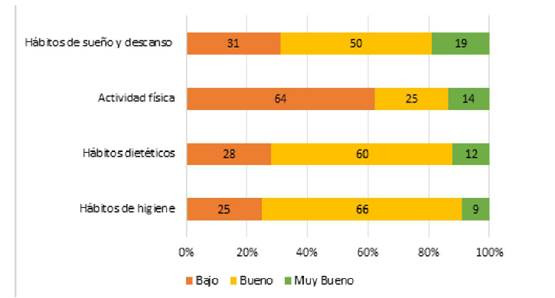
Distribución de la evaluación de las prácticas de autocuidado en los pacientes diabéticos.

De la misma manera, se determinó de acuerdo con las respuestas de los pacientes en las prácticas de autocuidado que presentan buenos hábitos de higiene en un 66% (66), el 60% (60) en hábitos dietéticos y un 50% (50) en hábitos de sueño y descanso. Sin embargo, los sujetos encuestados presentan un bajo autocuidado con un 61% (61) respecto a la realización de actividad física (Figura 1). Referente a las prácticas de autocuidado en general, teniendo en cuenta las anteriormente mencionadas, se evidenció que dichas prácticas están en un nivel bueno con un 64% (64) vs personas tiene un bajo autocuidado en un 36% (36).

**Tabla 2:** Prácticas de autocuidado en pacientes diabéticos.

Aspectos		%	n
Hábitos de Higiene			
Frecuencia baño	Diario	98,00	98
	Cada 3 días	2,00	2
Lubricación de piel	Ninguna	22,00	22
	Pomada	2,00	2
	Crema	65,00	65
	Aceites	11,00	11
Presencia de prurito	Si	25,00	25
	no	75,00	75
Piel seca	Si	39,00	39
	no	61,00	61
Cuidado de los pies	Si	90,00	90
	no	10,00	10
Revisa los pies	Si	82,00	82
	no	18,00	18
Hábitos Dietéticos			
Comidas al día	1-2 comidas	11,00	11
	3-4 comidas	83,00	83
	>5 comidas	6,00	6
Alimentos entre comidas	Si	65,00	65
	no	35,00	35
Aspectos		%	n
Agregar sal a las comidas	Nunca	32,00	32
	A veces	28,00	28
Tipo de alimentación	Siempre	40,00	40
	Naturales	98,00	98
	Enlatados	1,00	1
Realiza dieta	Si	43,00	43
	no	57,00	57
Orientación	Ninguno	6,00	6
	Médico general	46,00	46
	Médico especialista	46,00	46
	Enfermero	2,00	2
Consulta por nutrición	Si	49,00	49
	no	51,00	51
Periodo de consulta	Nunca	42,00	42
	Cada mes	10,00	10
	Cada 3 meses	16,00	16
	Cada 6 meses	14,00	14
	>1 año	18,00	18
Consumo bebidas	Si	25,00	25
azucaradas	no	75,00	75
Consumo carnes rojas	Nunca	16,00	16
	1 vez por semana	51,00	51
	Cada 3 días	26,00	26
	Diario	7,00	7
Consumo carne blanca	Nunca	7,00	7
	1 vez por semana	17,00	17
	Cada 3 días	44,00	44
	Diario	33,00	33
Consumo de lácteos	Nunca	27,00	27
	1 vez por semana	11,00	11
	Cada 3 días	27,00	27
	Diario	38,00	38
Consumo de pescado	Nunca	1,00	1
	1 vez por semana	29,00	29
	Cada 3 días	36,00	36
	Diario	34,00	34
Consumo de verduras	Nunca	6,00	6
	1 vez por semana	7,00	7
	Cada 3 días	10,00	10
	Diario	77,00	77
Consumo de frutas	Nunca	8,00	8
	1 vez por semana	8,00	8
	Cada 3 días	13,00	13
	Diario	71,00	71
Consumo de leguminosas	Nunca	10,00	10
	1 vez por semana	46,00	46
	Cada 3 días	26,00	26
	Diario	18,00	18
Activida Física			
Realiza actividad	Si	44,00	44
	no	56,00	56
Tipo de actividad física	No realiza	54,00	54
	Aeróbicos	45,00	45
	Anaeróbicos	1,00	1
Aspectos	Nunc	%	n
Frecuencia de actividada	54,00	54	
	1 vez a la semana	6,00	6
	2 veces a la semana	3,00	3
	Cada 3 días	9,00	9
	Diario	28,00	28
Tiempo de actividad física	Ningún tiempo	51,00	51
	30-40 minutos	35,00	35
	50-60 minutos	12,00	12
	>60 minutos	2,00	2
Se fatiga al realizar actividad	Si	41,00	41
	no	59,00	59
Hábitos de Sueño y Descanso			
Horas que duerme	2-5 horas	14,00	14
	6-8 horas	81,00	81
	>10 horas	5,00	5
Sensación de descanso al	Si	70,00	70
dormir	no	30,00	30
Siestas	Si	62,00	62
	no	38,00	38
Padece insomnio	Si	18,00	18
	no	82,00	82

### Aspectos relacionados con prácticas de autocuidado y variables sociodemográficas (análisis bivariado)

En el análisis bivariado se busca reconocer cual es la relación entre la variable dependiente de evaluación del autocuidado en relación con cada una de las prácticas de hábitos de higiene, hábitos dietéticos, hábitos de actividad física y hábitos de sueño y descanso, frente a las variables sociodemográficas y diagnóstico de la enfermedad. En este análisis, se encontró de acuerdo con las variables sociodemográficas y hábitos dietéticos, que se asoció significativamente con la edad (p = 0,015). Con relación a la actividad física se encontró una asociación significativa con la educación (p =0,006), el nivel socioeconómico (p =0,007) y la ocupación (p =0,026). Sin embargo, analizar la variable de años de diagnóstico de la diabetes mellitus, la primera y última dimensión, no se evidencio una asociación significativa, debido a que todas presentaron valores p mayor de 0,05 (Tabla 3).

**Tabla 3:** 

	Autocuidado de hábitos de sueño y descanso				Autocuidado de hábitos de higiene				Autocuidado de hábitos dietéticos				Autocuidado actividad física			
Características	Bajo % (n) 25	Bueno % (n) 66	Muy bueno % (n) 9	Valor P	Bajo % (n) 28	Bueno % (n) 60	Muy bueno % (n) 12	Valor P	Bajo % (n) 61	Bueno % (n) 25	Muy bueno % (n) 14	Valor P	Bajo % (n) 31	Bueno % (n) 50	Muy bueno % (n) 19	Valor P
Sexo				0,921				0,138				0,591				0.167
Femenino	68,00 (17)	63,64 (42)	66,67 (6)		50,00 (14)	71,67 (43)	3,33 (4)		68,85(42)	60,00 (15)	57,14(8)		77,42(24)	62,00 (31)	52,63 (10)	
Masculino	32,00 (8)	36,36 (24)	33,33 (3)		50,00 (14)	28,33 (17)	66,67 (8)		31,15(19)	40,00 (10)	42,86(6)		22,58(7)	38,00 (19)	47,37(9)	
Edad				0,170				0,015				0,691				0.633
Min/40 años	16,00 (4)	10,61 (7)	11,11 (1)		28,57(8)	5,00 (3)	8,33(1)		13,11(8)	8,00 (2)	8,00 (2)		12,90(4)	10,00 (5)	15,79(3)	
41 a 56 años	12,00 (3)	34,85 (23)	33,33 (3)		28,57(8)	33,33(20)	8,33(1)		26,23(16)	32,00 (8)	32,00 (8)		25,81(8)	36,00 (18)	15,79(3)	
57 a 72 años	64,00 (16)	34,85 (23)	44,44 (4)		39,29(11)	41,67(25)	58,33(7)		40,98(25)	44,00 (11)	50,00 (7)		45,16(14)	42,00 (21)	42,11(8)	
73/Max años	8,00 (2)	19,70 (13)	11,11 (1)		3,57(1)	20,00 (12)	25,00 (3)		19,67(12)	16,00 (4)	0 (0)		16,13(5)	12,00 (6)	26,32(5)	
Estado civil				0,256				0,891				0,386				0.826
Soltero/viudo/divorciado	44,00 (11)	27,27 (18)	22,22 (2)		32,14(9)	31,67 (19)	25,00(3)		36,07(22)	24,00 (6)	21,43(3)		29,03(9)	30,00 (15)	36,84(7)	
Unión libre/casado	56,00 (14)	72,73 (48)	72,73 (48)		67,86(19)	68,33(41)	75,00(9)		63,93(39)	76,00 (19)	78,57 (11)		70,97(22)	70,00 (35)	70,00 (35)	
Nivel educativo				0,249				0,207				0,006				0.921
Ninguno/primaria	64,00 (16)	48,48 (32)	22,22 (2)		35,71(10)	53,33(32)	66,67 (8)		63,93(39)	32,00 (8)	21,43 (3)		51,61(16)	50,00 (25)	50,00 (25)	
Secundaria	16,00 (4)	19,70 (13)	19,70 (13)		17,86(5)	21,67 (13)	8,33(1)		11,48(7)	32,00 (8)	28,57(4)		16,13(5)	18,00 (9)	26,32(5)	
Técnico/universitario	20,00 (5)	31,82 (21)	55,56 (5)		46,43(13)	25,00 (15)	25,00(3)		24,59(15)	36,00 (9)	50,00(7)		32,26(10)	32,00 (16)	26,32(5)	
Ocupación				0,076				0,401				0,026				0.657
No Trabaja	4,00 (1)	9,90 (6)	0 (0)		0 (0)	10,00 (6)	8,33(1)		9,94(6)	4,00 (1)	0(0)		3,23(1)	6,00 (3)	15,79(3)	
Ama de casa	48,00 (12)	51,52 (34)	22,22 (2)		35,71(10)	50,00 (30)	50,00 (30)		50,82(31)	48,00 (12)	35,71(5)		58,06(18)	42,00 (21)	47,37(9)	
Oficios varios	24,00 (6)	12,12 (8)	11,11 (1)		21,43(6)	13,33(8)	8,33(1)		19,67(12)	12,00 (3)	0 (0)		12,90(4)	18,00 (9)	10,53(2)	
Otro	24,00 (6)	26,46 (18)	66,67 (6)		42,86 (12)	26,67 (16)	16,67 (2)		19,57 (12)	36,00 (9)	64,29 (9)		25,81 (8)	34,00 (17)	26,31 (5)	
Nivel socioeconómico				0,408				0,082				0,007				0.491
Estrato 1	84,00 (21)	77,27 (51)	55,56 (5)		67,86(19)	85,00 (51)	58,33(7)		85,25 (52)	72,00 (18)	50,00 (7)		87,10(27)	72,00 (36)	73,68(14)	
Estrato 2	12,00 (3)	13,64 (9)	33,33 (3)		17,86(5)	10,00 (6)	33,33(4)		13,11 (8)	16,00 (4)	21,43 (3)		21,43 (3)	18,00 (9)	21,05(4)	
Estrato 3 y 4	4,00 (1)	9,09 (6)	11,11 (1)		14,28(4)	5,00(3)	8,34 (1)		1,64 (1)	12,00 (3)	28,57 (4)		6,45(2)	10,00 (5)	5,27(1)	
Años diagnostico DM2				0,661				0,397				0,932				0,090
<1 año	16,00 (4)	15,15 (10)	0 (0)		17,86 (5)	15,00 (9)	0 (0)		11,47 (7)	16,00 (4)	21,43 (3)		25,81 (8)	8,00 (4)	10,53 (2)	
1-5 años	36,00 (9)	33,33 (22)	22,22 (2)		39,28 (11)	26,67 (16)	50,00 (6)		36,07 (22)	28,00 (7)	28,00 (7)		22,58 (7)	34,00 (17)	34,00 (17)	
6-10 años	24,00 (6)	18,19 (12)	22,22 (2)		17,86 (5)	23,33 (14)	8,33 (1)		21,31 (13)	20,00 (5)	14,29 (2)		16,13 (5)	28,00 (14)	5,26 (1)	
>10 años	24,00 (6)	33,33 (22)	33,33 (22)		25,00 (7)	35,00 (21)	41,67 (5)		31,15 (19)	36,00 (9)	35,71 (5)		35,48 (11)	30,00 (15)	30,00 (15)	

### Análisis multivariado de las prácticas de autocuidado

Utilizando el método de selección intencionada de covariables (descrito en el apartado de métodos), donde se incluyeron todas las variables que en el análisis bivariado de la relación de la evaluación de las prácticas de autocuidado con variables sociodemográficas y año de diagnóstico de la enfermedad. Se construyó el modelo final a partir las variables obligatorias de acuerdo con la relación de estas con el desenlace según la literatura y otras variables que mostraron cambios significativos en las covariables representados en OR crudos. Se halló que los participantes con diabetes mellitus en la dimensión de hábitos de higiene que tienen edades mayores (OR: 0.22; IC 95% 0.02-2.57) y el estar en unión libre o casado (OR: 0.43; IC 95% 0.13-1.43). Además, el ser de sexo femenino en la dimensión de hábitos dietarios (OR: 0.20; IC 95% 0.03; 1.29) pero solo se presentó que el tener una edad entre 41 a mayor de 73 años están asociadas significativamente con valores P menores de 5%. Igualmente, el ser ama de casa con la actividad física (OR: 0.32; IC 95% 0.04-2.21) y tener de uno a más de diez años de haber sido diagnosticado con diabetes mellitus con el sueño y el descanso tienen una asociación protectora con tener malas prácticas de autocuidado.

Adicionalmente, se encontró asociaciones de riesgo para tener malas prácticas de autocuidado, el ser de sexo femenino (OR: 2.13; IC 95% 0.34-13.15), el no tener un estrato socioeconómico de 1 (OR: 2.32; IC 95% 0.18-29.72) en la dimensión de higiene. En cuanto a la dimensión dietaría, se presentó en cuanto al ser ama de casa (OR: 2.09; IC 95% 0.26-16.40) y el tener entre uno a cinco años de diagnóstico de la enfermedad (OR: 3.51; IC 95% 0.40-30.17). Además, el tener un nivel educativo de ninguno hasta primaria (OR: 15.44; IC 95% 2.78-85.48) se encontró con significancia estadística en la actividad física y por último en la dimensión de sueño o descanso con el sexo femenino (OR: 2.81; IC 95% 0.56-14.06). Finalmente, en la evaluación de la especificación de enlace nos indicó que el modelo incluye las variables necesarias para la predicción del desenlace y al evaluar la bondad de ajuste por medio del test de Hosmer y Lemeshov, los modelos son buenos (Tabla 4).

**Tabla 4:** OR crudo para para malas prácticas de autocuidado en pacientes con diabetes mellitus.

Variable	Hábitos de higiene ORC (IC 95%)	Hábitos dietéticos ORC (IC 95%)	Actividad física ORC (IC 95%)	Hábitos de sueño y descanso ORC (IC 95%)
Sexo				
Masculino	1	1	1	1
Femenino	2.13 (0.34; 13.15)	0.20 (0.03; 1.29)	1.44 (0.29; 7.22)	2.81 (0.56; 14.06)
Edad				
Min/40 años	1	1	1	1
41 a 56 años	0.24 (0.03; 1.84)	0.07 (0.00; 0.60)*	0.54 (0.08; 3.47)	0.81 (0.11; 5.77)
57 a 72 años	1.64 (0.24; 11.12)	0.04 (0.00; 0.42)*	0.27 (0.03; 2.13)	2.01 (0.24; 16.23)
73/Max años	0.22 (0.02; 2.57)	0.01 (0.00; 0.28)*	0.26 (0.02; 2.67)	1.77 (0.16; 18.64)
Estado civil				
Soltero/viudo/divorciado	1	1	1	1
Variable	Hábitos de higiene ORC (IC 95%)	Hábitos dietéticos ORC (IC 95%)	Actividad física ORC (IC 95%)	Hábitos de sueño y descanso ORC (IC 95%)
Unión libre/casado	0.43 (0.13; 1.43)	0.61 (0.18; 2.04)	0.35 (0.10; 1.20)	1.33 (0.44; 4.05)
Nivel educativo				
Ninguno/primaria	1.83 (0.31; 10.66)	1.13 (0.19; 6.50)	15.44 (2.78; 85.48)*	1.34 (0.24; 7.35)
Secundaria	1	1	1	1
Técnico/universitario	0.45 (0.07; 2.91)	2.04 (0.39; 10.44)	3.48 (0.80; 15.16)	1.59 (0.30; 8.19)
Ocupación				
Oficios varios	1	1	1	1
No trabaja	0.36 (0.02; 5.43)	1	2.61 (0.17; 39.37)	2.61 (0.17; 39.37)
Ama de casa	0.38 (0.04; 3.23)	2.09 (0.26; 16.40)	2.09 (0.26; 16.40)	1.18 (0.18; 7.82)
Otros	1.01 (0.16; 6.37)	0.76 (0.13; 4.34)	0.34 (0.05; 1.98)	0.34 (0.05; 1.98)
Nivel socioeconómico				
Estrato 1	2.32 (0.18; 29.72)	0.44 (0.06; 2.90)	8.35 (0.66; 104.74)	1.74 (0.22; 13.55)
Estrato 2	1.32 (0.07; 24.78)	0.37 (0.03; 4.10)	7.38 (0.40; 135.94)	0.53 (0.04; 6.93)
Estrato 3 y 4	1	1	1	1
Años diagnostico DM2				
<1 año	1	1	1	1
1-5 años	0.56 (0.07; 4.23)	0.56 (0.07; 4.23)	1.40 (0.21; 9.35)	0.14 (0.02; 0.89)
6-10 años	1.09 (0.12; 9.42)	1.09 (0.12; 9.42)	1.80 (0.24; 13.29)	1.80 (0.24; 13.29)
>10 años	0.34 (0.04; 2.73)	2.86 (0.26; 30.69)	1.11 (0.14; 8.60)	0.28 (0.04; 1.81)

Valor p<0.05* Nota: ORC (odds ratico crudo)

## Discusión

El desarrollo de este estudio se enmarcó en las prácticas de autocuidado en personas diabéticas. Debido, a que la DM2 es una patología de importancia, dado que esta, es un gran problema de salud pública, ya que a nivel mundial el número de personas con DM2 va en aumento^3^. Encontrándose, de acuerdo con los resultados de la encuesta en el apartado de las prácticas de autocuidado realizada a los pacientes, se logró determinar que la mayoría tenía un nivel bueno de prácticas de autocuidado en un 64,00%, frente a un nivel bajo de dichas prácticas en un 36,00%. De manera similar Jackson y cols^9^, determinaron en su estudio que la mayoría de la muestra tenían un nivel de conocimiento general alto sobre el autocuidado en un 79,50%, mientras que el resto 20,50% mostró un conocimiento de autocuidado general bajo. Resultados diferentes a los reportados por Soler y cols^7^, quienes estimaron que un 90,91% tenían ausencia de prácticas de autocuidado y un 9,09% si presentaban prácticas de autocuidado.

Igualmente, en cuanto a los hábitos de higiene se estimó que eran buenos en un 66,00%. Resultados similares a los del estudio de Arteaga y cols^8^, donde estimaron buenos hábitos de higiene en un 52,50%. Referente al cuidado de la piel en la muestra del presente estudio, se observó que estos no presentaban piel seca y no presencia de prurito en ninguna zona del cuerpo. En contraste en lo observado por Arteaga y cols^8^ que refirieron que la población estudiada si presenta piel seca en una proporción de 73,80%, presencia de descamación en un 35,50% y prurito en un 53,80%. Según la American Diabetes Association, la diabetes puede afectar cualquier parte del cuerpo, incluida la piel. Hasta el 33% de las personas con diabetes tienen en algún momento en la vida una afección a la piel causada o afectada por esta enfermedad, incluyendo infecciones con bacterias y con hongos, y picazón. Por otra parte, la piel seca es debido a los altos niveles de glucosa en la sangre que ocasionan deshidratación^23^.

Asimismo, se observó en el estudio que el cuidado de los pies lo realizaba la mayoría de los pacientes, mientras que en un estudio realizado por Ramírez y cols^11^, apreciaron que un 64,80% tiene unas prácticas de autocuidado para la prevención del pie diabético medianamente adecuada. El cuidado de los pies es importante, debido a que ayuda a prevenir complicaciones como úlceras, infecciones que pueden conllevar a la gangrena y a la necesidad de amputaciones, esto debido a otras complicaciones de la diabetes como son las afecciones en nervios y arterias. Por lo tanto, estas alteraciones vasculares favorecen la aparición de edema y de zonas mal vascularizadas que dificultan la curación de lesiones y heridas, y la neuropatía diabética provoca pérdida de la sensibilidad, atrofia muscular y dolor^24^.

Respecto a los hábitos dietéticos, se encontró que el 83,00% consumían 3-4 comidas a al día y solo el 65,00% manifestó que consumían alimentos entre comida, a diferencia del estudio de Forero y cols^25^, en su estudio se encontró que el 96,30% consumían alimentos 3 veces al día, y el 59,00% consumían alimento entre comidas. Además, sobre el uso de sal en las comidas, en el actual estudio el 40,00% refirió agregar sal a sus comidas, resultado contrario al estudio mencionado quienes manifestaron que el 92,00 % no adicionaba sal a sus preparaciones servidas.

Además, de acuerdo con los tipos de alimentos se evidenció un alto porcentaje de consumo de verduras a diario (77,00%), frutas (71,00%), la carne (51,00%) fue la principal fuente de proteína con un consumo de una vez a la semana, pollo (44,00%) y pescado (36,00%) cada 3 días. Además, los encuestados refirieron que consumen leguminosas una vez por semana y lácteos (38%) a diario. Sin embargo, Forero y cols^25^, obtuvieron resultados diferentes, en el cual el consumo de verduras en su población de estudio era baja con un 30,00% y frutas en un 21,00%, en cuanto a la fuente de proteína fue igual al ser la principal fuente de proteína las carnes de res o pollo en un 50,00%, el 75,00% de estos pacientes consumían pescado 3 veces al mes, con relación al consumo de lácteos el 82,00% manifestó no consumir leche descremada; de los restantes, el 68,00% la consumía entre una y dos veces al día.

El 66,00% tomaba leche entera; de ellos, el 57,00% la consumía una vez al día y, el 15,00%, dos veces. Según la evidencia científica, en los hábitos alimentarios, donde se consumen abundantes alimentos ricos en grasas de origen animal y pocos alimentos vegetales, representa un predictor significativo de obesidad en pacientes diabéticos. Por otra parte, el consumo de menos calorías totales, grasas saturadas, proteínas de origen animal y carbohidratos refinados, tendrá grandes beneficios en el control del índice glicémico^26^.

Igualmente, con relación al manejo nutricional 57,00% señaló no llevar una dieta específica para la enfermedad, se estima que el 51,00% de la población no asiste a consulta por nutrición, sobre la cual el estudio de Forero y cols^25^, obtuvieron datos negativos en el que solo el 8,90% había tenido en algún momento consulta con un profesional en nutrición. La efectividad en el tratamiento nutricional implica corresponsabilidad entre paciente y personal sanitario. El nutriólogo o nutricionista debe ser la principal fuente de información sobre una dieta adecuada para el paciente y, por otro lado, el paciente debe hacerse cargo de su propia salud^27^.

De acuerdo con el contexto anterior, es conveniente proponer la implementación de planes y programas orientados al manejo de la nutrición de esta población, que sea accesible y cuente con personal capacitado, para impactar de forma positiva y mejorar las conductas alimenticias que generan afecciones en la salud de estos, para así poder reducir complicaciones cardiovasculares y generar un mantenimiento de los niveles de glucemia en los rangos normales.

Por otro lado, con respecto a la actividad física un estudio de Arteaga y cols^8^, afirman que las personas con DM2 deben realizar ejercicio de forma regular, además, que en ellas es recomendable hacer actividades aeróbicas, de intensidad moderada, 150 minutos a la semana, distribuidos en tres días; con una dieta y peso adecuado, para evitar el riesgo de desarrollar complicaciones. En su investigación reportan que la actividad física no es realizada en un 77,50%.

Resultados semejantes se evidencian en el presente estudio, donde hay una mayor proporción de pacientes que no hacen actividad física, con un 56,00%. Finalmente, en relación con los hábitos de sueño y descanso, se demostró que en general los encuestados tienen buenos hábitos de descanso y sueño en un 50,00%, así como, Arteaga y cols^8^ estimaron que el 67,30% presentaron buenos hábitos de descanso y sueño. Es importante que las personas que padecen diabetes descansen sus horas completas, ya que la falta de descanso produce estrés, por lo que existe una alta posibilidad de que aumente la glicemia en sangre. Además, dormir en espacio de poco tiempo durante el día, ayuda a minimizar el estrés, y con ello disminuye la glicemia^28^.

Además, en el presente estudio en relación con el análisis bivariado, se encontró que la relación de las prácticas de autocuidado con los aspectos sociodemográficos existe una asociación entre edad con los hábitos dietéticos. Igualmente, el nivel socioeconómico, nivel educativo y ocupación con los hábitos de actividad física. Por otro lado, las prácticas de hábitos de higiene, descanso y sueño no fueron significativas con ninguna de las variables sociodemográficas del estudio. Otros estudios, han demostrado que se correlacionaban otras variables, por ejemplo, Jackson I y cols^9^ hallaron asociaciones entre el conocimiento del autocuidado con el nivel de educación (p = 0,000), los ingresos mensuales (p = 0,000) y la duración de la diabetes (p = 0,008).

Además, es probable que las mujeres tengan más conocimientos que los hombres; sin embargo, esto no alcanzó significación estadística (p = 0,655). Este estudio también mostro que los pacientes solteros tenían más conocimientos sobre el autocuidado, aunque no se alcanzó significación estadística (p =0,115). No se encontró asociación significativa entre conocimiento y edad, ocupación, tener familiares, parientes o amigos con diabetes, ser fumador actual o consumir alcohol.

Por otra parte, a la construcción del modelo final a través de la regresión logística se observó que la población a estudio presentó asociaciones protectoras para no tener malas prácticas de autocuidado en la dimensión de hábitos de higiene al tener una edad avanzada, el tener estado civil de casado o unión libre y con una formación de técnico o universitaria. Además, el ser de sexo femenino en la dimensión de hábitos dietarios y el no tener trabajo o ser ama de casa con la actividad física. Una posible explicación de la asociación de estas variables por ejemplo las personas con un nivel educativo y un nivel socioeconómico más alto tienen una mayor probabilidad de obtener conocimiento de los medios de comunicación, libros e internet. Además, tienen menos barreras en la comunicación con la atención médica. equipo y puede tener un buen conocimiento de la información^29^^,^^30^.

Por el contrario, la relación como factor de riesgo para presentar posibles complicaciones durante la enfermedad por un mal hábito de autocuidado se presentó al tener un estrato 1 en la dimensión de higiene personal, el haber sido diagnosticado de diabetes entre uno a cinco años en la dimensión de hábitos dietarios, el no tener ninguna educación o hasta primaria con la actividad física y ser del sexo femenino con el hábito de sueño o descanso.

En cuanto a las limitaciones del estudio, se pudo, presentar sesgo de información no diferencial, relacionado con la medición de las prácticas de autocuidado por medio del autoreporte, lo cual llevaría a un subregistro de este mismo, que se podría ver evidenciado en los resultados asociativos con una disminución de este, además, un mayor tamaño podría haber ayudado dar más poder para las medidas de asociación que por ahora se ven con tendencias y que aportaría para que los OR con sus IC95% disminuyan el rango. Sin embargo, dentro de sus fortalezas cabe resaltar que permitirá a los investigadores ampliar los conocimientos acerca la DM2, de igual manera aprender el diseño, estructura metodológica e investigativa de un proyecto de investigación.

Por otro lado, aporta más documentación bibliográfica que se tiene de la enfermedad, además, los resultados ayudarán a identificar realmente las necesidades de estos pacientes con respecto a las prácticas de autocuidado, para que así los entes gubernamentales involucrados en el proceso puedan implementar estrategias acordes a las necesidades reales de estos pacientes. Igualmente, la realización de futuros estudios con la inclusión de nuevas variables, correlacionar con otras escalas y utilizar otros diseños de investigación como los estudios de cohorte o de intervención que permitan tener una mayor validez.

## Conclusiones

Este estudio confirmó un nivel de autocuidado bueno en todos los pacientes que participaron en el estudio. Con respecto a las variables relacionadas con las prácticas de autocuidado, teniendo en cuenta las respuestas correctas de acuerdo al total en cada una de las dimensiones, se evidencio buenos hábitos de higiene en una proporción alta, igualmente gran parte de la población mostró buenos hábitos dietéticos, asimismo, presentaron buenos hábitos de sueño y descanso en gran medida, de forma diferente se estimó en la dimensión de actividad física, en la cual se observó un bajo autocuidado con respecto a la realización de ejercicio. Donde, solo se encontró relación estadísticamente significativa entre la variable nivel de estudio, nivel socioeconómico y ocupación, frente a la variable de hábitos de actividad física y también se encontró significancia entre la variable de hábitos dietéticos con relación a la edad.

Además, los resultados alcanzados en esta investigación mostraron que los pacientes con DM2 en general indicaron tener buenas prácticas de autocuidado, sin embargo, se evidenció que un pequeño porcentaje consumía azúcar, alcohol y cigarrillo, y alimentos grasos. Lo cual indica que no tienen condiciones adecuadas para el control óptimo de la enfermedad y prevención de las complicaciones. Por lo tanto, es importante la realización de futuros estudios de la temática, debido a la escasez de estudios sobre de las prácticas de autocuidado en los pacientes diabéticos, ya que es esencial identificarlas para llevar a cabo intervenciones oportunas que disminuyan y prevengan el riesgo de complicaciones y enfermedades desarrollado de la misma.
